# How does emotional insecurity affect non-suicidal self-injury among Chinese early adolescents: a longitudinal study

**DOI:** 10.1186/s13034-024-00839-4

**Published:** 2024-11-14

**Authors:** Xingcan Ni, Qiao Liang, Xiaoyan Liao, Huahua Wang, Chengfu Yu

**Affiliations:** 1 Department of Psychology and Research Center of Adolescent Psychology and Behavior, School of Education, Guangzhou University, Guangzhou, 510006 Guangdong China; 2 School of Psychology, South China Normal University, Guangzhou, 510631 Guangdong China

**Keywords:** Emotional insecurity, Non-suicidal self-injury (NSSI), Peer exclusion, School climate

## Abstract

**Background:**

Non-suicidal self-injury (NSSI) is a serious public health concern. Emotional insecurity is a crucial predictor of NSSI among adolescents. However, few studies have elucidated the specific mechanisms between emotional insecurity and NSSI.

**Methods:**

This study employed a longitudinal research design, using a sample of 886 Chinese early adolescents (*M*_age_ at T1 = 10.62 years, *SD* = 0.77 years; 47.40% females), and conducted two surveys six months apart to examine the mediating role of peer exclusion between emotional insecurity and NSSI, as well as the moderating effect of school climate.

**Results:**

The results indicated that peer exclusion significantly mediated the connection between emotional insecurity and adolescent NSSI. Moreover, school climate significantly moderated the connection between emotional insecurity and peer exclusion. Specifically, the impact of emotional insecurity on peer exclusion was significant only in adolescents who reported a negative school climate, but non-significant in those who reported a positive school climate.

**Conclusions:**

These findings provide a robust theoretical foundation and practical insights to help inform the prevention of and interventions for NSSI in adolescents.

## Background

Non-suicidal self-injury (NSSI) is conventionally defined as purposeful damage to one’s own body tissue without the intention of dying [1]. A recent study conducted in China reported a 17.6% prevalence of NSSI among early adolescents over a six-month period [2]. Given the substantial challenges that NSSI poses to adolescents’ physical and mental well-being [3, 4], it has garnered increasing attention from researchers.

Numerous factors contribute to the complex etiology of NSSI. Among these, family factors are thought to be one of the crucial determinants of NSSI in adolescents [5]. Emotional security is an important dimension for measuring family relationships. Adolescents who lack emotional security in their parents’ relationship (i.e., they experience emotional insecurity) are likely to develop a range of psychological and behavioral issues [6, 7]. Emotional insecurity arises from negative emotional experiences triggered by interparental conflict and inappropriate parenting practices [8], which causes individuals to develop avoidant responses to conflict situations [9]. According to the experience avoidance model [10], NSSI serves as a coping strategy for emotional avoidance. When faced with emotional insecurity induced by interparental conflict, adolescents may turn to NSSI to regulate their adverse affective experiences. Empirical research has also indicated that adolescents who are exposed to interparental conflict long-term tend to have higher levels of emotional insecurity and are more prone to NSSI [11, 12]. However, past research has mostly explored emotional insecurity as an important mediating variable between interparental conflict and adolescent problem behaviors, with limited understanding of the underlying mechanisms between emotional insecurity and NSSI. Moreover, studies on the connection between emotional insecurity and NSSI have remained at the psychological level, with less exploration from the perspective of interpersonal relationships. Therefore, examining the impact of emotional insecurity on adolescent NSSI from an interpersonal viewpoint may provide more targeted theoretical insights and practical guidance for promoting the healthy growth of adolescents.

Emotional security theory [9] posits that emotional security plays a crucial role in emotional regulation and behavioral responses, and a lack of emotional security is often linked to developmental challenges in children’s and adolescents’ social adaptability [12, 13]. A previous study demonstrated that those who experienced higher degrees of emotional insecurity typically had worse social skills and struggled to establish and maintain positive interpersonal relationships [14]. Peer exclusion, a negative peer relationship experience, refers to an individual being subjected to various forms of exclusion by peers, such as being rejected or ignored [15]. Peer exclusion is a significant risk factor for NSSI [16, 17]. When adolescents experience emotional insecurity, they often present heightened anxiety and social avoidance during interactions with their peers [18]. Such social interactions make it difficult for them to form healthy peer relationships, thereby increasing the likelihood of peer exclusion [14, 19]. Faced with these negative interpersonal experiences, adolescents often lack effective coping strategies and may protect themselves by resorting to negative defense mechanisms and emotional regulation methods [20, 21], such as engaging in NSSI [16, 17].

Although no empirical research has directly demonstrated how peer exclusion mediates the connection between emotional insecurity and NSSI, some studies have offered indirect evidence. For instance, Zhang et al. [22] discovered that parental psychological control and social maladjustment among adolescents were mediated by campus exclusion. Similarly, Zhao et al. [23] reported that peer victimization significantly mediated the relationship between childhood trauma and adolescent NSSI. These studies demonstrate how adverse parenting practices and maladaptive issues are mediated by negative peer relationship experiences such as peer exclusion. Therefore, we propose the following hypothesis:

### Hypothesis 1

Peer exclusion will mediate the relationship between emotional insecurity and NSSI among adolescents.

Not all adolescents with a high degree of emotional insecurity experience the same degree of peer exclusion or NSSI. According to the stress-buffering hypothesis [24], protective resources can compensate and alleviate the negative effects of adverse family environments on adolescent development. As they age, adolescents tend to spend less time in family life and more time in school life, highlighting the importance of the school climate for understanding and subsequently preventing the emergence of maladaptive behaviors [25]. School climate refers to the relatively long-lasting and stable characteristics of the school environment that can be experienced by its members and that significantly shape their psychology and behavior [26]. Studies suggest that a positive school climate can buffer the impact of a dysfunctional family on adolescent development [27, 28]. In addition, students who perceive a positive school climate are more likely to report experiencing positive interpersonal relationships [29]. If adolescents experience a supportive school atmosphere, they may achieve a sense of security and emotional support from their peers and teachers that is lacking in their home life [30]. Positive interactions with school members reduces the incidence of peer exclusion [31]. Therefore, a favorable school climate may promote adolescents’ participation in social activities, reduce avoidance behaviors stemming from emotional insecurity, and consequently reduce the likelihood of peer exclusion. Therefore, we propose the following hypothesis:

### Hypothesis 2

School climate will moderate the indirect association between emotional insecurity and NSSI via peer exclusion. Specifically, adolescents who report a more favorable school climate will exhibit a substantially weaker relationship between emotional insecurity and peer exclusion.

**Fig. 1:**
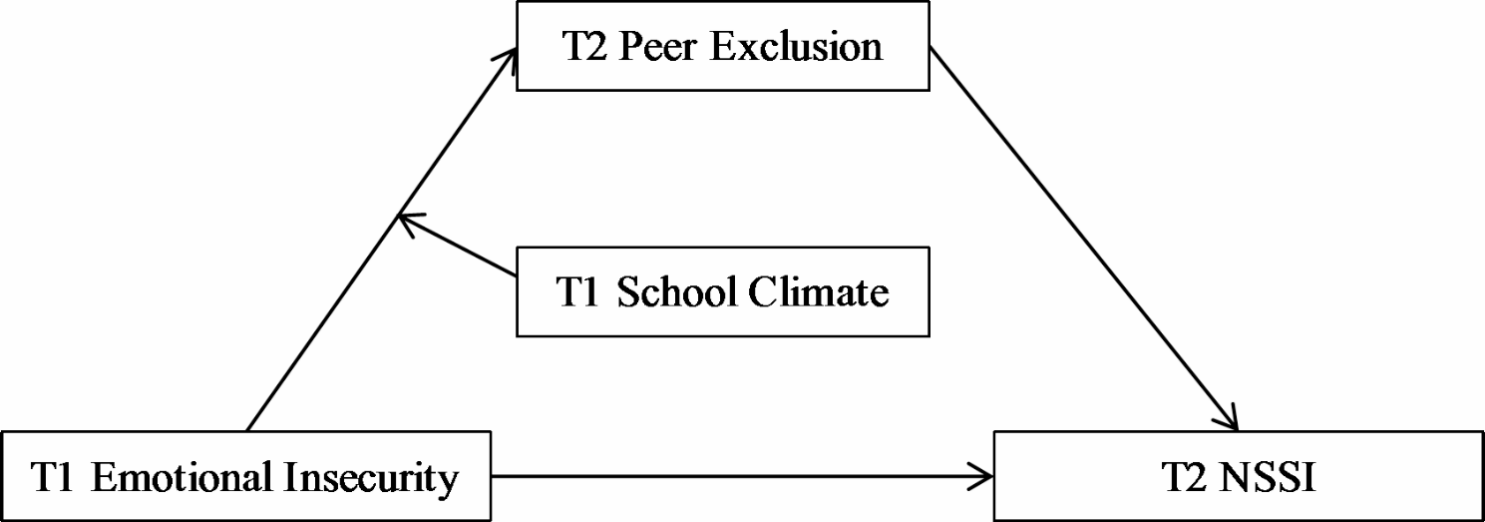
The proposed mediated moderation model between emotional insecurity and NSSI. *NSSI* Non-suicidal self-injury

Through a two-wave longitudinal design with a six-month gap between assessments, this study investigated the mediating role of peer exclusion, as well as the moderating role of school climate, in the association between emotional insecurity and adolescent NSSI. Figure 1 illustrates the proposed model, which offers an explanatory mechanism for this link.

## Methods

### Participants

In this study, randomized cluster sampling was employed to select participants from four standard primary schools located in Guangdong Province, China, for a two-wave longitudinal study with a six-month interval between waves. At Time 1 (T1), the participants completed questionnaires containing measures of emotional insecurity, school climate, NSSI, and demographic information (i.e., gender and age). At Time 2 (T2), the participants completed questionnaires containing measures of peer exclusion and NSSI. The final dataset comprised 886 valid questionnaires, of which 466 were from males (52.60%) and 420 were from females (47.40%), with an average age at T1 of 10.62 years (*SD* = 0.77; range from 9 to 14 years).

### Measures

#### Emotional insecurity

Emotional insecurity was measured using the Emotional Reactivity Subscale in Security in the Interparental Subsystem [32]. The scale consists of nine items scored on a four-point scale (1 = *not at all true of me* to 4 = *very true of me*). An example item includes, “When my parents argue, I feel upset.” A higher average score indicates greater emotional insecurity. Cronbach’s α coefficient at T1 was 0.88.

#### NSSI

NSSI was measured using the Chinese version of the NSSI questionnaire [33], adapted from the Deliberate Self-Harm Inventory [34]. The scale consists of twelve items scored on a six-point scale (1 = *never* to 6 = *several times a week*). An example item includes, “In the past six months, have you deliberately cut yourself but without suicidal intent?” A higher average score indicates greater NSSI. Cronbach’s α coefficient at T1 was 0.85 and at T2 was 0.90.

#### Peer exclusion

Peer exclusion was measured using the Peer Exclusion Scale, as adapted by Xu and Niu [35]. The scale consists of six items scored on a five-point scale (1 = *strongly disagree* to 5 = *strongly agree*). An example item includes, “It sometimes feels as though some peers seem to be ignoring me.” A higher average score indicates a greater degree of peer exclusion. Cronbach’s α coefficient at T2 was 0.96.

#### School climate

School climate was assessed using the School Climate Scale [36]. The questionnaire consists of 25 items scored on a four-point scale (1 = *never* to 4 = *always*). An example item includes, “In our school, I can tell my teachers about my problems and difficulties.” A higher average score indicates a more positive student perception of the school climate. Cronbach’s α coefficient at T1 was 0.89.

#### Demographic covariates

Age and gender were taken into account as demographic factors in all of the analyses because prior research has shown a substantial correlation between these variables and NSSI [37–39].

### Procedure

The Ethics Committee of Guangzhou University (GZHU202351) approved this study. With informed consent from parents and students, a trained psychology research assistant helped the participants complete the self-reported questionnaire. The entire process lasted for approximately 30 min. The participants’ anonymity was guaranteed, and they were informed that they can withdraw from the study at any time without consequence. As a token of appreciation, participants received a commemorative pen upon completion of the questionnaire.

### Data analysis

First, descriptive statistics and correlational analyses were generated using IBM^®^ SPSS^®^ Statistics Version 27.0 (IBM Corp., Armonk, NY, USA). Second, the SPSS PROCESS macro model 4 [40] was employed to examine the longitudinal mediation model. Third, the SPSS PROCESS macro model 7 [40] was employed to examine the longitudinal moderated mediation model. We further explored the interactions using simple slopes analysis. To ascertain the significance of the effects, a bootstrapping procedure with bias correction was applied based on a sample size of 5000; an effect was considered statistically significant if the 95% confidence interval (CI) did not include zero. Throughout all analytical stages, gender, age, and T1 NSSI were controlled for as covariates.

## Results

### Descriptive statistics and correlation analysis

Among all participants, 25.73% (*N* = 228) reported at least one NSSI at T1, and 21.11% (*N* = 187) reported at least one NSSI at T2. The means, standard deviations, and correlations between the measured variables are displayed in Table 1. As expected, T1 emotional insecurity, T2 peer exclusion, T1 NSSI, and T2 NSSI were significantly positively correlated. T1 school climate was significantly and negatively related to these four variables.

**Table 1:** Descriptive statistics and correlations for all variables

Variables	1	2	3	4	5	6	7
1.T1 EI	1.00						
2.T2 PE	0.19***	1.00					
3.T1 SC	−0.09**	−0.31***	1.00				
4.T1 NSSI	0.20***	0.21***	−0.16***	1.00			
5.T2 NSSI	0.18***	0.23***	−0.08*	0.65***	1.00		
6.Gender	0.08*	0.18***	0.06	0.02	0.04	1.00	
7.Age	−0.02	0.01	−0.01	0.02	0.04	−0.09**	1.00
*Mean*	1.94	1.68	2.80	0.14	0.13	−	10.62
*SD*	0.72	1.00	0.53	0.39	0.43	−	0.77

Gender was coded as 1 = males and 2 = females

*EI* Emotional insecurity, *NSSI* Non-suicidal self-injury, *PE* Peer exclusion, *SC* School climate, *T1* Time 1, *T2* Time 2

*p < 0.05, **p < 0.01, ***p < 0.001

### The mediation model

**Fig. 2:**
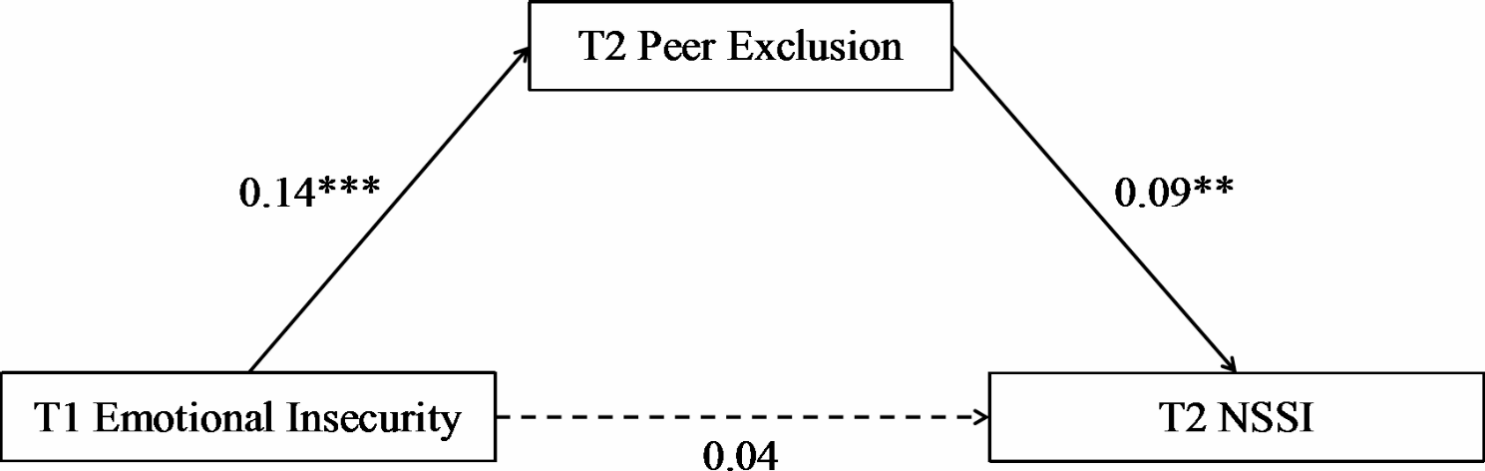
The mediating effect of peer exclusion in the relationship between emotional insecurity and NSSI. *NSSI* Non-suicidal self-injury. **p < 0.01, *** *p* < 0.001

The results of the longitudinal mediation model are illustrated in Fig. 2. T1 emotional insecurity positively predicted T2 peer exclusion (*β* = 0.14, *p* < 0.001), but did not significantly predict T2 NSSI (*β* = 0.04, *p* > 0.05). Furthermore, T2 peer exclusion positively predicted T2 NSSI (*β* = 0.09, *p* < 0.01). The bootstrapping analyses revealed that T2 peer exclusion had a mediation effect of 0.01 (*SE* = 0.01, 95% CI [0.00, 0.03]), thus supporting H1. Details are shown in Table 2.

**Table 2:** Results of the mediation model for the effect of emotional insecurity on NSSI

	Model 1 (T2 PE)				Model 2 (T2 NSSI)			
	β	SE	t	95% CI	β	SE	t	95% CI
Age	0.03	0.04	0.62	[− 0.06, 0.11]	0.04	0.03	1.19	[− 0.03, 0.10]
Gender	0.33	0.06	5.11***	[0.20, 0.46]	0.04	0.05	0.69	[− 0.07, 0.14]
T1 NSSI	0.18	0.03	5.48***	[0.12, 0.24]	0.62	0.03	23.53***	[0.57, 0.67]
T1 EI	0.14	0.03	4.14***	[0.07, 0.20]	0.04	0.03	1.35	[− 0.02, 0.09]
T2 PE					0.09	0.03	3.30**	[0.04, 0.14]
*R* ^2^	0.09				0.43			
*F*	22.41***				133.57***			

Gender was coded as 1 = males and 2 = females

*EI* Emotional insecurity, *NSSI* Non-suicidal self-injury, *PE* Peer exclusion, *T1* Time 1, *T2* Time 2

**p < 0.01, ***p < 0.001

### The moderated mediation model

**Fig. 3:**
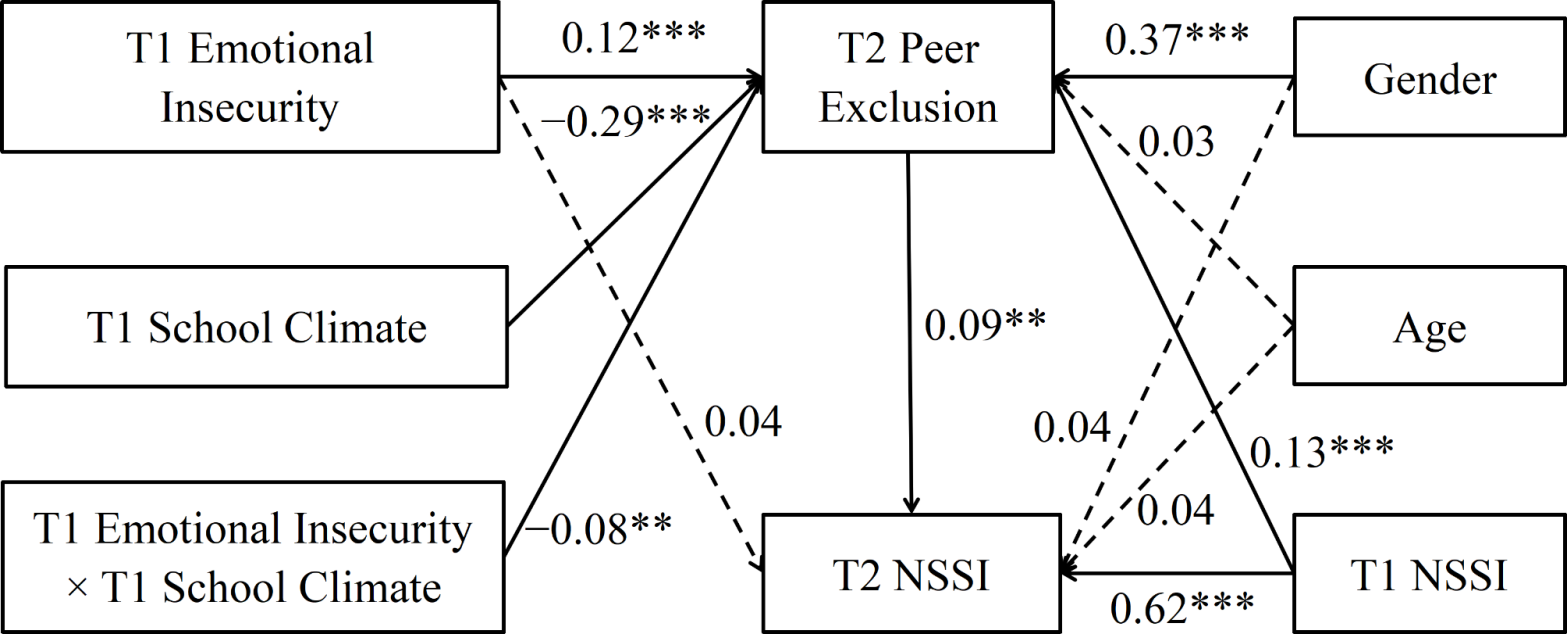
Test of the longitudinal moderated mediation model. *NSSI* Non-suicidal self-injury. **p < 0.01, ***p < 0.001

The results of the longitudinal moderated mediation model are illustrated in Fig. 3. The interaction between T1 emotional insecurity and T1 school climate had a significant effect on T2 peer exclusion (*β* = −0.08, *p* < 0.01). This indicates that T1 school climate moderated the connection between T1 emotional insecurity and T2 peer exclusion; hence, Hypothesis 2 was supported. Details are shown in Table 3.

**Fig. 4:**
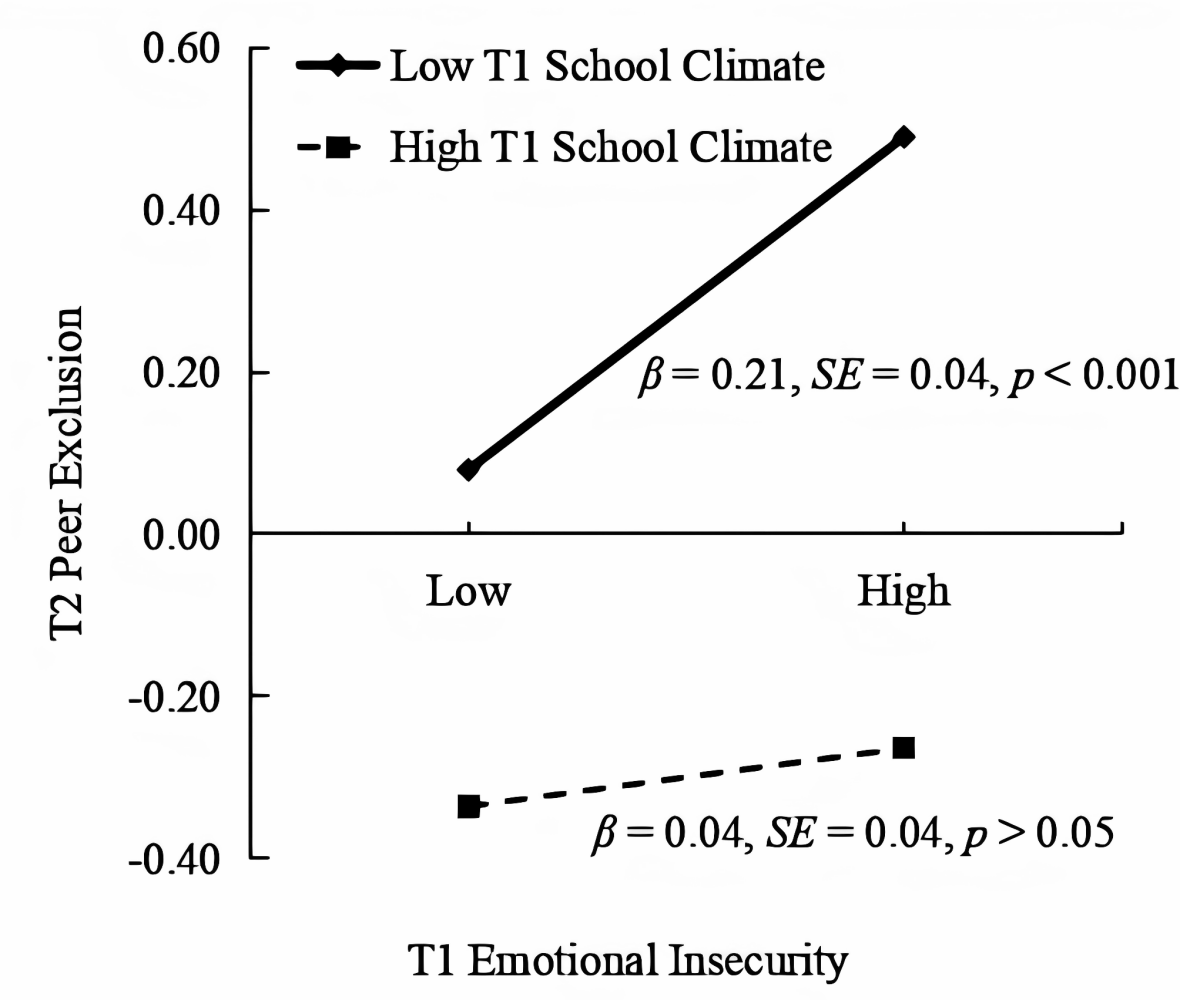
Interactive effect of school climate and emotional insecurity on peer exclusion

Subsequently, simple slopes analysis revealed that the impact of T1 emotional insecurity on T2 peer exclusion was only significant in adolescents with low T1 school climate (*β* = 0.21, *p* < 0.001), but non-significant in those with high T1 school climate (*β* = 0.04, *p* > 0.05; see Fig. 4). Bootstrapping analyses revealed that the mediating effect of T2 peer exclusion in the pathway from T1 emotional insecurity to T2 NSSI was only significant in adolescents with low T1 school climate (*Effect* = 0.02, *SE* = 0.01, 95% CI [0.01, 0.04]; see Table 4), but non-significant in those with high T1 school climate (*Effect* = 0.01, *SE* = 0.01, 95% CI [− 0.01, 0.01]). That is, perception of a positive school climate undermined the impact of emotional insecurity on peer exclusion.

**Table 3:** Results of the moderated mediation model for the effect of emotional insecurity on NSSI

	Model 1 (T2 PE)				Model 2 (T2 NSSI)			
	β	SE	t	95% CI	β	SE	t	95% CI
Age	0.03	0.04	0.63	[− 0.05, 0.10]	0.04	0.03	1.19	[− 0.03, 0.10]
Gender	0.37	0.06	5.93***	[0.25, 0.49]	0.04	0.05	0.69	[− 0.07, − 0.14]
T1 NSSI	0.13	0.03	4.10***	[0.07, 0.19]	0.62	0.03	23.53***	[0.57, 0.67]
T1 EI	0.12	0.03	3.83***	[0.06, 0.18]	0.04	0.03	1.35	[− 0.02, 0.09]
T1SC	−0.29	0.03	−9.33***	[− 0.35, − 0.23]				
T1 EI × T1 SC	−0.08	0.03	−2.77**	[− 0.14, − 0.02]				
T2 PE					0.09	0.03	3.30**	[0.04, 0.14]
*R* ^2^	0.18				0.43			
*F*	31.50***				133.57***			

Gender was coded as 1 = males and 2 = females

*EI* Emotional insecurity, *NSSI* Non-suicidal self-injury, *PE* Peer exclusion, *SC* School climate, *T1* Time 1, *T2* Time 2

**p < 0.01, ***p < 0.001

**Table 4:** Conditional indirect effects of T1 emotional insecurity on T2 NSSI via T2 peer exclusion by levels of T1 school climate

Levels of T1 school climate	Effect size	Boot SE	95% CI
1. Low (*M*-*SD*)	0.02	0.01	[0.01, 0.04]
2. Med (*M*)	0.01	0.01	[0.01, 0.03]
3. High (*M + SD*)	0.01	0.01	[− 0.01, 0.01]

*NSSI* Non-suicidal self-injury, *T1* Time 1, *T2* Time 2

## Discussion

In this longitudinal study of Chinese early adolescents, emotional insecurity predicted NSSI through the mediation of peer exclusion. Additionally, the first half of the model, namely the association between emotional insecurity and peer exclusion, was moderated by school climate. Specifically, a positive school climate emerged as a protective element that reduced peer exclusion among adolescents with emotional insecurity.

Consistent with Hypothesis 1, peer exclusion significantly mediated the connection between emotional insecurity and NSSI. Previous research indicates that peer exclusion is a crucial mechanism mediating the links between adverse parenting practices and maladaptive issues [22]. This study extends previous research by revealing a developmental pathway from emotional insecurity to peer exclusion, and then to NSSI. It also confirms a classic theory in psychology, namely ecological systems theory: one system (the family system) can influence adolescent development through another system (the peer system) [41]. A stable and positive emotional environment plays a crucial role during adolescent development [13]. According to emotional security theory [9], when adolescents experience emotional insecurity due to interparental conflict, they may exhibit a range of negative psychological and behavioral responses, such as sensitivity, depression, and avoidance [42]. These responses can affect adolescents’ social interactions, causing a series of challenges to their social adaptation that may prevent them from forming healthy peer relationships [19, 43]. Adolescents facing peer exclusion during social interactions are prone to emotional distress and a sense of thwarted belongingness [44]. They are often unaware of how to deal with these adverse interpersonal experiences and negative emotions and may resort to maladaptive coping mechanisms, such as NSSI, to alleviate their psychological pain and facilitate an escape from reality [16, 17]. In the current study, the direct impact of emotional insecurity on NSSI was not statistically significant, in contrast to the findings of an earlier study [11]. This may be attributed to the timing of NSSI measurements, as previous studies employed cross-sectional designs. A recent longitudinal study supports this viewpoint [12]. In this recent study, emotional insecurity at Time 1 did not directly affect NSSI at Time 2, but instead it indirectly influenced NSSI at Time 2 through depressive symptoms. This finding suggests that the direct influence of emotional insecurity on NSSI may be temporary; emotional security must exert an impact on subsequent social adaptation in order affect NSSI several months later.

Consistent with Hypothesis 2, school climate moderated the impact of emotional insecurity on peer exclusion. Specifically, emotional insecurity significantly predicted an increase in peer exclusion but only among adolescents who perceived a negative school climate. This finding is consistent with the stress-buffering hypothesis [24] and previous research [27, 28], indicating that school climate can buffer the impact of adverse family environments on adolescent development. This finding suggests that a positive school climate can protect adolescents from peer exclusion even if they experience emotional insecurity due to interparental conflict. One possible explanation for this is that adolescents’ inclusive tendencies can be shaped by a positive school climate. Adolescents are more inclined to demonstrate understanding, welcomeness, and inclusiveness towards their peers in a positive school climate [45, 46]. Therefore, even when interacting with adolescents who exhibit sensitivity and avoidance due to emotional insecurity, they can understand and accommodate them, which helps reduce the occurrence of peer exclusion. In addition, a positive and supportive school climate provides adolescents with a sense of safety and belonging [30, 47]. When adolescents feel accepted and respected at school, this can alleviate anxiety and avoidance behaviors stemming from emotional insecurity, which promotes positive interactions with peers and helps adolescents establish healthy peer relationships [31].

Although many previous studies indicate that the occurrence of NSSI among adolescents varies by gender and age [37–39], such differences were not observed in our study. This lack of variation may be attributed to the specific developmental stage of our research participants: our sample focused on early adolescents, while most previous studies targeted mid-to-late adolescent groups. Some studies support this viewpoint. For instance, Steinhoff et al. [48] and Wilkinson et al. [49] noted that gender differences in NSSI only emerge during mid-to-late adolescence rather than the early stages. However, our study did reveal gender differences in peer exclusion. Compared to male adolescents, female adolescents are more likely to experience peer exclusion. Some previous studies support this finding [50]. Prior research indicates that female adolescents place greater emphasis on friendships and are often more sensitive to peer relationships than male adolescents [51], which may lead to a higher likelihood of female adolescents reporting experiences of peer exclusion.

This study has some limitations that warrant mentioning. First, the variables examined in this study were subject to adolescent self-reporting, which could be compromised by social desirability bias. Future studies should seek more objective measurements. Peer reports, teacher reports, and experimental studies could be employed to replicate these findings. Second, the sample population of this study was drawn from four primary schools in Guangdong Province, which does not offer broad representation. Future studies could select regions with different economic development levels and cultural environments and examine horizontal comparisons between different types of schools to enhance the specificity and external validity of the research findings. Third, our study only investigated the protective function of school climate in the connection between emotional insecurity and peer exclusion. According to the stress-buffering hypothesis [24], other factors may mitigate the impact of emotional insecurity and/or peer exclusion on adolescent NSSI. For instance, positive interpersonal factors (e.g. social support), intrinsic psychological resources (e.g. self-compassion, sense of meaning in life), and adaptive emotional regulation strategies (e.g. cognitive reappraisal) have been shown to help adolescents cope adaptively with negative life events and reduce their harmful effects [52–55]. Future research should investigate the protective function of these factors in altering the strength of the relationships among emotional insecurity, peer exclusion, and NSSI. Fourth, data from just two time points were considered in this investigation. Multi-wave longitudinal data may be used in future research to investigate the correlations between variables.

Despite these limitations, our study has substantial significance for the prevention of NSSI and interventions for the treatment of NSSI in adolescents. Theoretically, our study is the first to explore the collective effects of emotional insecurity, peer exclusion, and school climate on NSSI among adolescents. It not only revealed the intrinsic connection between emotional issues stemming from adverse family environments and NSSI but also provides theoretical guidance for the prevention and intervention strategies that address NSSI in adolescents, expanding the application of emotional security theory within the realm of pediatric NSSI. From a practical perspective, to reduce the incidence of NSSI in adolescents, the following measures are suggested. First, parents should endeavor to minimize interparental conflict and foster a warm and supportive home environment to enhance adolescents’ emotional security. To achieve this goal, parents and family members can participate in family therapy, which can effectively enhance friendly communication within the family and create a supportive family environment [56]. In addition, a four-session Family Communication Program has been shown to enhances adolescents’ emotional security by encouraging healthy marital dispute resolution techniques [57]. Second, parents and educators should pay attention to the peer interaction issues of adolescents, assist them in developing better social skills, and teach them to value individual differences and build and sustain positive interpersonal connections. Third, schools should create a positive, equitable, and inclusive educational environment. In the school environment, it is crucial to establish peer group norms and implement comprehensive anti-exclusion programs. Taking these steps can help adolescents address peer exclusion issues effectively, thereby reducing the likelihood of their NSSI [58]. Lastly, in the process of preventing and intervening NSSI, adolescents from dysfunctional family environments or those who have experienced peer exclusion should be the focus of special attention. Educators, parents, and mental health professionals should guide these adolescents in expressing and venting negative emotions appropriately through mental health education and other similar initiatives. Adolescents in more severe situations can employ cognitive-behavioral therapy techniques, mindfulness-based practices, and emotional regulation training to help them better cope with the negative cognitions and emotions resulting from adverse interpersonal experiences [59].

## Conclusion

Our two-wave longitudinal study reveals that peer exclusion significantly mediated the relationship between emotional insecurity and NSSI. Moreover, a positive school climate buffers the negative impact of emotional insecurity and peer exclusion. These findings suggest that focusing on reducing peer exclusion and fostering a positive family and school environment may reduce NSSI among early adolescents. Based on the findings of this study, future research can further explore the underlying mechanisms of NSSI in early adolescents and propose more comprehensive and targeted programs for the prevention and intervention of NSSI in this age group.

## Data Availability

The datasets used and/or analysed during the current study are available from the corresponding author on reasonable request.
